# Desertification risk fuels spatial polarization in ‘affected’ and ‘unaffected’ landscapes in Italy

**DOI:** 10.1038/s41598-021-04638-1

**Published:** 2022-01-14

**Authors:** Samaneh Sadat Nickayin, Rosa Coluzzi, Alvaro Marucci, Leonardo Bianchini, Luca Salvati, Pavel Cudlin, Vito Imbrenda

**Affiliations:** 1 Planning and Design Faculty, Agricultural University of Iceland, 311 Hvanneyri, Borgarbyggð, Iceland; 2 Institute of Methodologies for Environmental Analysis, National Research Council (IMAA-CNR), Contrada Santa Loja, 85050 Tito Scalo, Italy; 3 Department of Agricultural and Forestry Sciences (DAFNE), Tuscia University, Via San Camillo de Lellis, 01100 Viterbo, Italy; 4 Department of Economics and Law, University of Macerata, Via Armaroli 43, 62100 Macerata, Italy; 5 Global Change Research Institute (CAS), Lipova 9, 37005 Ceske Budejovice, Czech Republic

**Keywords:** Climate-change ecology, Ecological modelling, Ecosystem ecology, Ecology, Environmental sciences, Environmental social sciences, Natural hazards

## Abstract

Southern Europe is a hotspot for desertification risk because of the intimate impact of soil deterioration, landscape transformations, rising human pressure, and climate change. In this context, large-scale empirical analyses linking landscape fragmentation with desertification risk assume that increasing levels of land vulnerability to degradation are associated with significant changes in landscape structure. Using a traditional approach of landscape ecology, this study evaluates the spatial structure of a simulated landscape based on different levels of vulnerability to land degradation using 15 metrics calculated at three time points (early-1960s, early-1990s, early-2010s) in Italy. While the (average) level of land vulnerability increased over time almost in all Italian regions, vulnerable landscapes demonstrated to be increasingly fragmented, as far as the number of homogeneous patches and mean patch size are concerned. The spatial balance in affected and unaffected areas—typically observed in the 1960s—was progressively replaced with an intrinsically disordered landscape, and this process was more intense in regions exposed to higher (and increasing) levels of land degradation. The spread of larger land patches exposed to intrinsic degradation brings to important consequences since (1) the rising number of hotspots may increase the probability of local-scale degradation processes, and (2) the buffering effect of neighbouring (unaffected) land can be less effective on bigger hotspots, promoting a downward spiral toward desertification.

## Introduction

The interplay of natural and human processes leveraged soil degradation, drought, and desertification risk in both affluent countries and emerging economies, causing a global reduction in land productivity^1–4^. Global warming, economic growth, and population increase were recognized as basic drivers of large-scale land degradation, a phenomenon affecting more than 40 per cent of the Earth’s surface—including parts of Europe, the United States, and Australia^5–7^. While climate aridity is still regarded as a basic driver of land degradation, human pressure is playing a more significant role than in the past (e.g.^8–10^). In the Mediterranean basin—one of the world hotspot for soil degradation—desertification risk driven by population growth, crop intensification, urban expansion, and soil pollution, was frequently associated with biophysical factors such as climate aridity, soil quality, and poor vegetation cover^11–17^. All these factors produce potentially devastating effects on the environment^18–20^—not tied exclusively to the later Holocene (e.g.^21,22^).

Assuming land vulnerability to degradation as a dynamic, multi-faceted attribute of ecosystems^23^, a permanent monitoring of landscape trajectories may provide the necessary background to understand the intimate linkage between landscape configuration and the intrinsic mechanisms and processes of land degradation^24–26^. Since degradation of (natural) ecosystems may lead to landscape fragmentation, patch fragmentation and landscape complexity were sometimes regarded as basic indicators of land vulnerability to degradation^27–29^. While characterizing the structural state of a landscape, fragmentation involves a series of processes—such as loss of a given habitat type, increase in isolation, greater exposure to human pressure along fragment edges—activating changes in the function of the remaining fragments^30–32^. However, few studies have investigated this issue in a specific land degradation perspective. Lin et al.^33^ found that, as vegetation patches became more fragmented and homogeneous, soils become more exposed to desertification risk. Whether these processes lead to land degradation, in turn triggering complex transformations responsible for changes in landscape composition, has been occasionally explored (e.g.^34^).

A quantitative characterization of spatial patterns is a crucial step when assessing the linkage between landscape structure and the underlying ecological processes related to land degradation^35–37^. A dashboard of landscape-level metrics was adopted in the present study with the aim at verifying the intrinsic relationship between landscape fragmentation and land degradation in Italy. In more detail, the working hypothesis is that a given territorial system may undergo different patterns of land vulnerability to degradation, depending on the various dynamics related to fragmentation of the land patches exposed to different levels of vulnerability^38^. For this purpose, the Environmental Sensitive Area (ESA) methodology, developed in the framework of the EU-funded MEDALUS (Mediterranean Desertification and Land Use) international project and largely applied in Mediterranean socioeconomic contexts, represents an effective monitoring system of the level of land vulnerability to degradation^39^.

Based on these premises, our study was articulated in two steps: (1) Italian land was classified into three vulnerability levels (‘unaffected’, ‘fragile’, and ‘critical’) according with the Environmentally Sensitive Area (ESA) nomenclature, as a function of four key dimensions of land degradation (climate, soil quality, vegetation cover, and human pressure); (2) composition, configuration, and structure of a ‘simulated landscape’ constituted of the three ESA vulnerability classes were studied in Italy using 15 metrics producing indicators aggregated at the spatial level of administrative regions, a relevant domain for environmental reporting and policy implementation. ‘Critical’ areas have been considered as ‘vulnerability hotspots’ requiring specific mitigation measures^40^. Assuming responses to land degradation as based on a set of land management actions dependent on the local context^41^, the present study assesses the evolution of landscape metrics referring to a ‘simulated landscape’ based on land degradation vulnerability in Italy between the early-1960s and the early-2010s. In line with these perspectives, regional mitigation plans may promote a policy shift from driver-specific and process-specific targets to a more comprehensive set of practical actions mixing responses adapted to the local context.

## Methodology

### Study area

**Figure 1:**
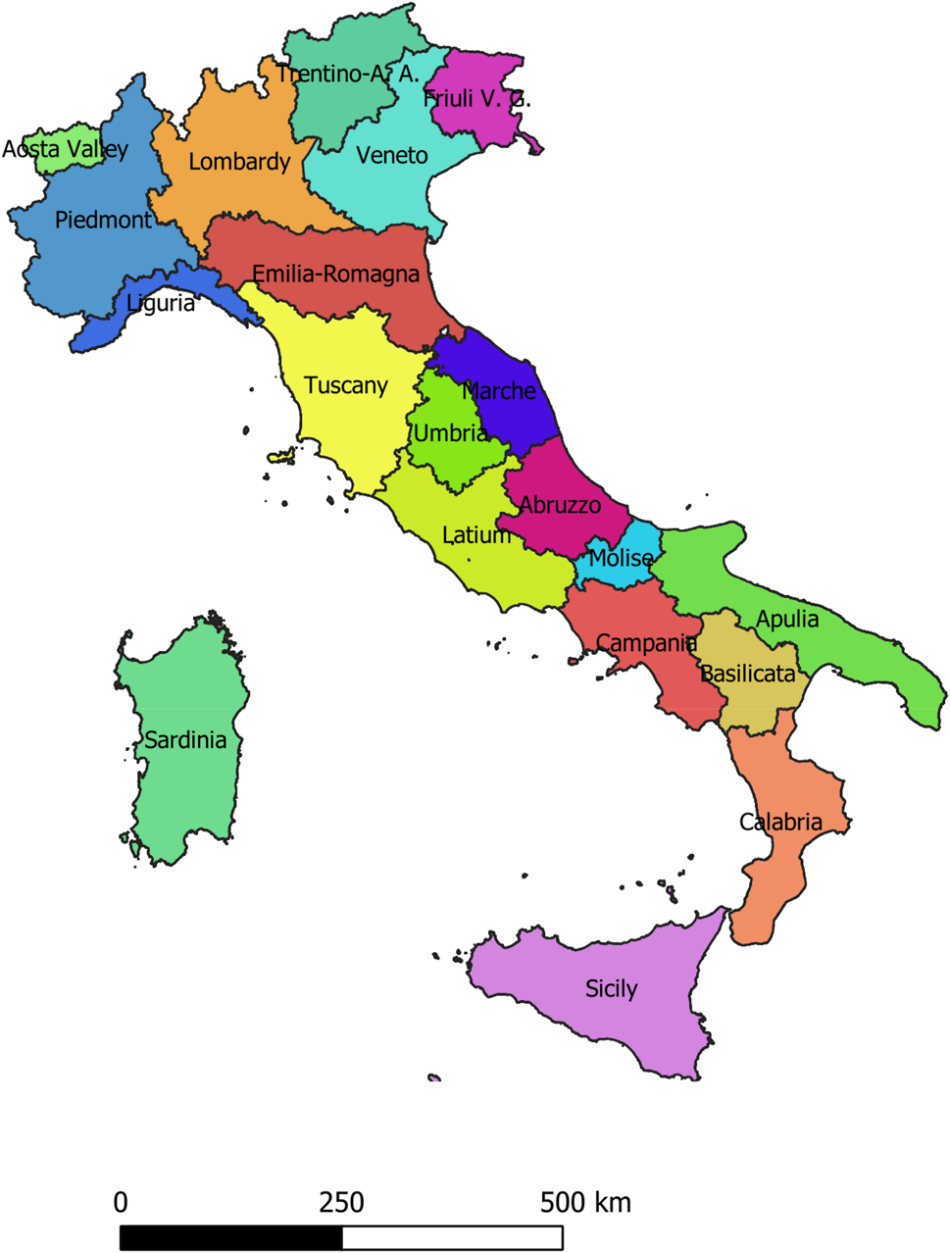
A stylized map of Italy with regional boundaries (this map was created using the Italian statistical atlas of municipalities, a free application downloaded from www.istat.it).

Italy is positioned at the heart of the Mediterranean Basin reaching an impressive extension of coastline of about 7600 kms. The Apennine mountain chain cuts through the Italian Peninsula, while the Alps separate it from the rest of Europe. Italy is partitioned in three macro-areas (North, Centre, South) and 20 administrative regions (Fig. 1) for a total area of about 300,000 km^2^, in turn subdivided in three elevation classes (35% mountains, 42% uplands, 23% lowlands:^6^. Large part of Italy enjoys a Mediterranean climate with mild-cool winter and dry-warm summer. Rainfalls typically increase with elevation whereas temperatures display the reverse pattern^42^. A distinctive trait of Italy is the socioeconomic gap between Northern (more affluent) and Southern (more disadvantaged) regions reflected in the asymmetric distribution of population, settlements, and natural resources over space^43^. Due to these specific conditions, Italy is an intriguing case when analysing the complex relationship between biophysical and socioeconomic variables and their influence on land vulnerability to degradation.

### The ESA approach

The Environmentally Sensitive Area (ESA) is a model developed within the MEDALUS (Mediterranean Desertification And Land Use) international research project^44^ to identify areas prone to desertification through the use of a composite index (hereafter, the ESAI). ESA methodology is likely the most popular and flexible indicator‐based scheme to estimate vulnerability to land degradation^25^ having extensively validated on the field and with independent indicators of land degradation, estimated in different bio-geographical contexts (see^19^. Lastly, the ESAI was demonstrated to be a stable and reliable index, being little influenced by spatial and temporal heterogeneity of the composing indicators^45^. To estimate land degradation vulnerability, the standard ESA model accounts for four components: climate quality, soil quality, vegetation quality, and human factors/land management quality (Table 1). The thematic layers used in this work are the most reliable, updated, and referenced data currently used for ESAI assessments in Italy^46^. We covered a time window of 50 years by estimating land degradation vulnerability at three years (1960, 1990, 2010), the only available dates to fully develop the model at national level (Table 2).

**Table 1:** List of landscape metrics assessing the spatial distribution of land vulnerability to degradation in Italy.

Acronym	Metric	Rationale
MPS	Mean patch size	The arithmetic mean of the patch sizes
PSCoV	MPS Coefficient of variation	The coefficient of variation in patch size relative to the mean patch size
ED	Edge density	The sum of the lengths of all edge segments, divided by the total area
MSI	Mean shape index	The average perimeter-to-area ratio for weighted by the size of its patches
AWMSI	Area-weighted mean shape index	The average shape index of patches, weighted by patch area
MPFD	Mean patch fractal dimension	The sum of 2 times the logarithm of patch perimeter divided by the logarithm of patch area for each patch of the corresponding patch type, divided by the number of patches of the same type
AWMPFD	Area-weighted mean fractal dimension	The average patch fractal dimension, weighted by patch area
LPI	Largest patch index	The percent of the landscape or class that the largest patch comprises
LSI	Landscape shape index	The sum of the landscape boundary and all edge segments within the landscape boundary divided by the square root of the total landscape area
SDI	Shannon diversity index	Minus the sum, across all patch types, of the proportional abundance of each patch type multiplied by that proportion
SHEI	Shannon evenness index	The observed Shannon’s Diversity Index divided by the maximum Shannon’s Diversity Index for that number of patch types
SIEI	Simpson’s Evenness Index	The observed Simpson’s Diversity Index divided by the maximum Simpson’s Diversity Index for that number of patch types
MSIEI	Modified Simpson’s evenness index	The observed modified Simpson’s diversity index divided by the maximum modified Simpson’s diversity index for that number of patch types
MPI	Mean proximity index	The degree of isolation and fragmentation of the corresponding patch type
MNN	Mean nearest neigh	The shortest straight-line distance between the focal patch and its nearest neighbor of the same class
IJI	Interspersion index and juxtaposition index	The observed interspersion divided by maximum possible interspersion for the given number of patch types

**Table 2:** Selected variables describing the spatial configuration of the Italian landscape based on three vulnerability classes (‘unaffected’, ‘fragile’, ‘critical’) by administrative region and year (the ID code of each region is reported in brackets).

Region	Number of patches			Mean patch size (km^2^)			Vulnerability level (ESAI)		
	1960	1990	2010	1960	1990	2010	1960	1990	2010
Piedmont (1)	900	1195	1260	27.2	20.0	18.8	1.315	1.319	1.331
Aosta Valley (2)	76	173	199	38.1	16.1	13.9	1.289	1.270	1.301
Lombardy (3)	692	973	918	31.7	20.8	21.8	1.326	1.340	1.369
Trentino A.A. (4)	413	690	721	31.3	18.4	17.6	1.273	1.262	1.291
Veneto (5)	606	511	485	27.9	30.9	32.2	1.321	1.347	1.367
Friuli V.G. (6)	235	266	264	30.7	26.4	26.3	1.294	1.296	1.304
Liguria (7)	250	348	340	20.4	14.3	14.7	1.314	1.300	1.313
Emilia-Romagna (8)	752	796	674	28.6	26.2	30.6	1.345	1.370	1.390
Tuscany (9)	775	1141	902	29.0	19.1	24.0	1.331	1.338	1.361
Umbria (10)	364	504	482	22.8	15.9	16.6	1.296	1.309	1.318
Marche (11)	340	521	518	27.1	17.2	17.3	1.332	1.365	1.369
Latium (12)	771	664	687	21.5	23.9	23.0	1.338	1.351	1.357
Abruzzo (13)	339	509	502	31.4	20.6	20.8	1.338	1.360	1.325
Molise (14)	202	216	224	21.6	20.2	19.5	1.359	1.384	1.361
Campania (15)	512	659	655	25.7	19.1	19.1	1.338	1.361	1.360
Apulia (16)	527	463	527	35.1	38.6	33.8	1.392	1.428	1.404
Basilicata (17)	317	431	455	31.2	22.7	21.4	1.370	1.385	1.382
Calabria (18)	572	723	815	25.6	20.0	17.6	1.326	1.342	1.334
Sicily (19)	657	751	748	37.3	31.5	31.7	1.434	1.427	1.431
Sardinia (20)	605	654	642	37.8	34.5	35.0	1.367	1.377	1.387

### Environmental variables and thematic indicators

Climate quality has been analysed by considering three variables: average annual rainfall rate, aridity index, and aspect^47^ computed by using data from the Agro-meteorological Database of the Italian Ministry of Agriculture (including nearly 3,000 weather stations providing daily records since 1951; technical details available in^46^. For the soil dimension, considered as a quasi-static factor due to its very slow changes over time^48^, we extracted the required information to generate the standard ESA elementary layers (depth, texture, slope, and parent material) from the European Soil Database (Joint Research Centre, JRC) at 1 km^2^ pixel resolution^8^ and from other ancillary sources: (a) the Italian ‘Map of the water capacity in agricultural soils’ (Italian Ministry of Agriculture, see^46^,(b the Ecopedological and Geological maps of Italy (realized by JRC and the Italian Geological Service), and (c) a land system map produced by the National Centre of Soil Cartography (Florence, Italy).

Vegetation quality includes four variables: plant cover, fire risk, erosion protection, and drought resistance. These parameters were evaluated using the sequence of CORINE Land Cover (CLC) maps for the years 1990 (CLC90) and 2012 (CLC12) (https://land.copernicus.eu/pan-european/corine-land-cover), and a CORINE-like ‘Topographic and Land Cover Map of Italy’^40^ produced by the National Research Council (CNR) and the Italian Touring Club (TCI) in 1960 (LUM60). The CLC nomenclature encompasses 44 land cover classes grouped into a three-level hierarchy. Similarly, LUM60 is a standard 1:200,000 map covering Italy with a nomenclature of 22 classes that is compatible with the CLC hierarchical system^49^.

Land management quality includes indicators accounting for population dynamics and specific changes in land-use^20^. In particular, human pressure was estimated with indicators of density and annual growth rate of resident population (ISTAT 2006). Lastly, starting from the abovementioned maps (LUM60, CLC90, CLC12), an indicator of land-use intensity was computed that groups the considered land cover classes according to their use intensity and the potential level of vulnerability to degradation. Full explanations of the calculation of these indicators are available in Salvati et al. (2013).

### The composite index of land vulnerability to degradation

Following Bajocco et al.^8^, we applied a scoring system based on the documented linkage between each variable and land degradation phenomena. The adopted system was derived from Recanatesi et al.^49^. ESA model relies on the calculation of four quality indicators related to climate (Climate Quality Index, CQI), soil (Soil Quality Index, SQI), vegetation (Vegetation Quality Index, VQI), and land management (Land Management Quality Index, MQI). Each of them was computed as the geometric mean of the different scores associated to every input variable. To combine them easily, values of each quality indicator were classified adopting a standard score ranging from 1 (very low vulnerability to degradation) to 2 (very high vulnerability to degradation), assigning equal weight to each layer^8^. The final ESAI was computed as the geometric mean of the four quality indicators. Italy was classified into three classes corresponding to different levels of vulnerability^50^: (1) areas unaffected (or only potentially affected) by land degradation (ESAI < 1.225), (2) ‘fragile’ areas (1.225 < ESAI < 1.375), and (3) ‘critical’ areas (ESAI > 1.375). The spatial resolution of the produced maps was 1 km^2^, which is coherent with the resolution of the single layers^46^.

### Statistical analysis

Following Recanatesi et al.^49^, the ESAI values were treated as a ratio variable since they range continuously from 1 to 2 over large sample sizes. In particular, we estimated the average ESAI score at the three investigated years by using the 20 administrative regions of Italy as the elementary analysis’ domain. This country’s partition is consistent with the characteristics and resolution of the indicators selected. In these regards, the Italian National Action Plan (NAP) to Combat Desertification has designed the twenty administrative regions as the effective spatial unit to coordinate and implement mitigation policies. Indicators proposed in the present study are therefore useful for the identification of strategies contrasting land degradation that can be implemented in the Regional Action Plans (RAPs), a spatial planning tool that each regional administration developed in line with the guidelines of the NAP^51^. The average ESAI score was calculated at each spatial unit using the ‘zonal statistics’ procedure developed in ArcGIS (ESRI Inc., Redwoods, USA). This procedure computes a surface-weighted average of the ESAI (i.e., recorded on each elementary pixel) belonging to the spatial unit being analysed^48^.

A total of 15 landscape metrics (Table 1) assessing patch size, fragmentation, shape, fractality, and juxtaposition, were chosen with the aim at providing a comprehensive assessment of the Italian landscape’s spatial configuration over time^52^. These metrics were derived from the above-mentioned ESA maps using simple computational tools from ArcGIS and ‘Patch Analyst’ package, well suited to a vast audience of planners and stakeholders^46^. Selected landscape metrics were reported at the regional scale using descriptive statistics. Pair-wise relationships between each metric and the ESAI average value were analysed at the regional scale using Spearman non-parametric rank coefficients testing for significance at *p* < 0.05 after Bonferroni’s correction for multiple comparisons^53^. A Principal Component Analysis (PCA) was carried out at the same spatial scale considering together 16 variables (15 landscape metrics and the ESAI average value) separately at three years (1960, 1990, 2010). Components with eigenvalue > 1 were identified and evaluated considering together the position of loadings (variables) and scores (administrative regions) within a biplot. The PCA was used to represent the latent relationship between landscape structure and the level of land vulnerability at an aggregated spatial scale (administrative regions) in Italy, removing (or containing) redundancy among individual variables in the sample^54^.

## Results

### Spatio-temporal trends in landscape metrics and the ESAI

The average ESAI in Italy increased by 1.5% (rising from 1.34 in 1960 to 1.36 in 2010) and delineates worse conditions toward land degradation vulnerability all over the country. The rank of the most vulnerable regions (i.e. Sicily and Apulia, both located in Southern Italy) was rather stable during the study period. From the third position downwards, the ranking changed rapidly in the study period. Basilicata (Southern Italy) ranked third in the early-1960s and dropped to the fifth position in the early-2010s. Emilia Romagna (Northern Italy) ranked sixth in the early-1960s and only third in the early-2010s. As a general trend, Northern Italian regions showed larger increases in the ESAI than those recorded in Southern Italy. Following the increase in the level of vulnerability to land degradation in Italy (Table 2), more fragmented landscape patches were observed in all Italian regions in the first observation interval (1960–1990), except for Veneto, Latium, and Apulia. A more heterogeneous trend was observed in the second period (1990–2010): an increase in the number of landscape patches was observed in 8 regions; the reverse trend was observed in 12 regions. A continuous increase in the number of patches in both time periods was observed in Northern Italy (Piedmont, Aosta Valley, Trentino Alto Adige, Friuli Venezia Giulia), and in Southern Italian regions affected by a moderate level of land vulnerability (Molise, Calabria and, in part, Basilicata). Veneto was the only region showing the reverse trend, with a continuous reduction in the number of land patches. Faced with these dynamics, the average size of land patches systematically decreased in Italy, with the exception of Veneto. In 1960, Aosta Valley, Apulia, Sicily, and Sardinia were the regions with the biggest (average) patch size. This ranking changed in the early-1990s, as only Sardinia confirmed the largest average size, preceded by Apulia and followed by Veneto. In the early-2010s, Sardinia was confirmed as the region with the largest and least fragmented land patches, followed by Apulia, Veneto, and Emilia Romagna.

### Correlation analysis

Results of a non-parametric correlation analysis between selected landscape metrics and the ESAI (Table 3) delineate a substantially different correlation profile between the three times under study, in turn highlighting how landscape structure—thanks to the differential arrangement of the ESAI classes (‘unaffected’, ‘fragile’, ‘critical’) over space—was associated with the average level of land vulnerability to degradation. In particular, the level of regional vulnerability in the early-1960s increased significantly with the mean proximity index and with indicators of landscape diversification (SIEI and MSIEI). Conversely, the level of vulnerability decreased with the mean nearest neighbour index and with the ‘interspersion and juxtaposition’ index. The early-1990s represented a transitional context, with the level of vulnerability increasing moderately with average patch size and decreasing with edge density. In the early-2010s, the strength of the relationship between the level of vulnerability and mean patch size consolidated. At the same time, a positive and significant relationship between land vulnerability and mean proximity index was observed. These results highlight the latent linkage between the average level of vulnerability and landscape structure on a regional scale. In the early-1960s, the most vulnerable landscapes showed greater fragmentation and diversification, alternating ‘fragile’ and ‘critical’ patches with ‘unaffected’ patches. In the subsequent periods, and especially in the early-2010s, more homogeneous landscapes with bigger class patches (mostly ‘fragile’ or ‘critical’), were exposed to a higher level of land vulnerability to degradation.

**Table 3:** Pair-wise Spearman rank correlations between the average level of vulnerability to land degradation (regional ESAI, see Table 2) and landscape metrics (see Table 1 for acronyms) at the same spatial scale (only significant coefficients at *p* < 0.05 were shown after Bonferroni’s correction for multiple comparisons).

Variable	1960	1990	2010
MPI	0.63		0.71
MNN	− 0.75		
IJI	− 0.88		
MPS		0.61	0.69
ED		− 0.70	
LPI	− 0.63		
SIEI	0.71		
MSIEI	0.71		

### Multivariate analysis

Principal Component Analysis confirms the results obtained in the correlation analysis (see above) and provides further indications about the intrinsic characteristics of administrative regions with respect to the landscape structure and the overall level of vulnerability to land degradation. Extraction of the principal components (Table 4) identifies, for all the study years, four components that together explain more than 90% of the overall matrix variance.

**Table 4:** Results (loadings) of a Principal Component Analysis run on the full set of landscape metrics (see Table 1 for acronyms) considered in this study at the regional scale in Italy, by year.

Metric	Component 1			Component 2			Component 3			Component 4		
	1960	1990	2010	1960	1990	2010	1960	1990	2010	1960	1990	2010
ESAI	0.60				0.62	0.65						
MPI				0.79	0.92	0.77						
MNN	− 0.67				0.61							0.87
IJI		0.77	0.67	− 0.61								
MPS				0.76	0.86	0.80						
PSCoV						− 0.64	0.92	0.86	0.69			
ED	0.71	0.75	0.83	− 0.62								
MSI							− 0.68				0.67	
AWMSI	0.69							0.80	0.69			
AWMPFD	0.75				0.66			0.63				
LPI	− 0.64	− 0.78	− 0.71			− 0.64						
LSI	0.75					0.69			0.65			
SDI	0.84	0.98	0.92									
SHEI	0.84	0.98	0.92									
SIEI	0.97	0.94	0.81									
MSIEI	0.96	0.94	0.84									
Expl. Var.%	46.7	38.4	40.0	21.9	26.4	30.1	18.6	17.9	15.1	6.6	7.5	6.7

#### Exploring latent landscape structures at the first observation time (early-1960s)

**Figure 2:**
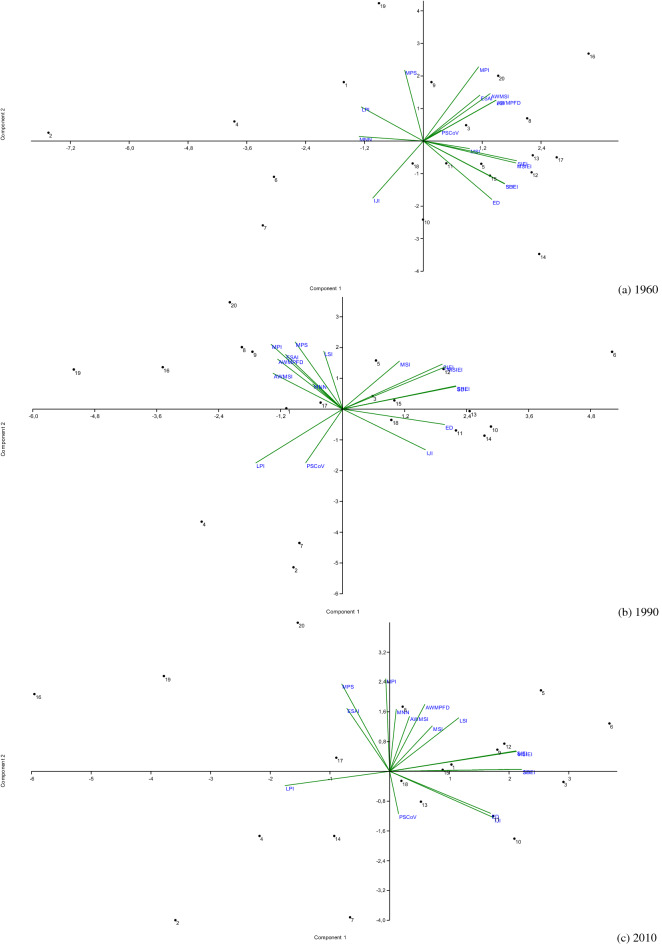
Biplot of a Principal Component Analysis outlining latent relationships between landscape metrics and the level of land vulnerability to degradation (regional codes were reported in Table 2).

In the early-1960s, Component 1 (46.7%) was positively associated with the average ESAI at the regional scale and selected indicators of landscape fractality, diversification, and heterogeneity (MPI, ED, AWMSI, AWMPFD, LSI, SDI, SHEI, SIEI and MSIEI). At the same time, Component 1 was associated negatively with the presence of big, homogeneous patches (LPI) and with the average distance from the nearest land patch (MNN). Component 2 (21.9%) outlines a gradient of landscape vulnerability, assigning positive loadings to indicators of landscape homogeneity (MPS) and patch distance (MPI), and negative loadings to the indicators of fractality (ED) and interspersion (IJI). Component 3 (18.6%) outlines a landscape fragmentation gradient, assigning positive loadings to PSCoV index and negative loadings to MSI. Finally, Component 4 (6.6%) was not associated with any landscape indicator (Fig. 2).

#### Exploring latent landscape structures at the second observation time (early-1990s)

The four components extracted for the early-1990s explained 90.2% of the overall variance and break down the landscape structure into different gradients from what has been observed for the early-1960s. For instance, the ESAI was associated with Component 2 – and no longer with Component 1; Component 4 was instead associated with a specific landscape dimension. Specifically, Component 1 (38.4%) highlights a landscape fragmentation gradient, with indicators of diversification, fractality and juxtaposition between patches (SDI, SHEI, SIEI, MSIEI, ED) receiving positive loadings. The associated LPI index evidencing more homogeneous landscapes (i.e. indicating bigger patches of the same vulnerability class) received a negative loading. Component 2 (26.4%) outlines a positive association between the ESAI and four landscape metrics (MPI, MNN, MPS, AWMPFD). These metrics, however, provided heterogeneous indications on the relationship between landscape structure and land degradation, as they highlight (1) a significant relationship between average patch size (MPS) and the (average) level of land vulnerability, as well as (2) a more intrinsic relationship between dispersion and fractal indices (MPI, MNN, AWMPFD). These results may highlight a latent transformation from a fragmented and diversified distribution of vulnerability classes over space to a more homogeneous model. Landscape structure became more fractal and convoluted following the expansion of 'critical' areas, the growing fragmentation of ‘fragile’ areas, and the complexification of the spatial configuration of unaffected areas, which had represented, at least in the early-1960s, a relatively homogeneous landscape containing the expansion of ‘critical areas’ into surrounding land. Component 3 (17.9%) represents a landscape dispersion gradient (PSCoV, AWMSI, LSI) substantially decoupled from the level of land vulnerability. Component 4 (7.5%) was uniquely associated with the MSI index.

#### Exploring latent landscape structures at the third observation time (early-2010s)

Results of a Principal Component Analysis applied to landscape metrics in the early-2010s delineated an even more complex picture. The first four components extracted 91.9% of the overall variance, outlining a multivariate relationship between metrics and land vulnerability similar to what has been already observed in the early-1990s. In particular, Component 1 (40%) identified a landscape diversification gradient (positive loadings assigned to IJI, ED, SDI, SHEI, SIEI, MSIEI; negative loadings assigned to MPI and LPI). Component 2 (30.1%) outlined a direct relationship between land vulnerability and landscape homogenization (positive loadings of ESAI, MPS, MPI, MSI, AWMPFD, LSI; negative loading of PSCoV). Component 3 (15.1%) represent a gradient of landscape fractalization (PSCoV, AWMSI, LSI), mostly decoupled from the average level of land vulnerability. Finally, Component 4 (6.7%) was exclusively associated with the MNN metric.

#### Summary results of principal component analysis

The statistical distribution of component scores showed a different distribution of the Italian regions over time. In the early-1960s, Component 1 distinguished the regions with the highest level of land vulnerability in Southern Italy (associated with positive and higher scores) from mostly unaffected regions of Northern Italy, that were associated with negative values. This trend became more heterogeneous in the early-1990s, as the first two components included both Southern and Northern Italian regions classified with a comparatively high level of land vulnerability. The biplot referring to the early-2010s finally delineated a more pronounced spatial polarization between vulnerable regions positioned in the fourth quadrant (Apulia, Sardinia, Sicily and, in part, Basilicata, all placed in Southern Italy), and the other Italian regions placed in the remaining quadrants.

## Discussion

Using a traditional approach of landscape ecology, the present study evaluates the intimate structure of landscapes at different levels of vulnerability to land degradation using a wide set of metrics calculated at three time points in Italy. The diachronic analysis was developed through classical metrics that analyse structure and conformation of landscapes based on three different levels of vulnerability to land degradation, computationally treated as independent land-use classes. While the average level of land vulnerability increased significantly between the early-1960s and the early-2010s almost in all Italian regions^55^, landscape demonstrated to be increasingly fragmented, as far as the number of homogeneous patches and the mean patch size are concerned. The empirical results of a multivariate analysis confirm that the increase in the level of land vulnerability on a large scale has been associated with a structural change in the configuration of Italian landscapes^56^. The traditional polarization in severely affected and unaffected areas observed in the early-1960s was progressively replaced with an intrinsically disordered landscape intermixing (bigger) patches classified as ‘critical’ or ‘fragile’ land with (smaller) patches of land classified as ‘unaffected’ (e.g.^57–59^. On average, this process was more intense in regions exposed to a higher level of land degradation.

At the beginning of the study period, regions with a high level of degradation were associated with diversified landscapes characterized by a spatial balance between different classes of land vulnerability. Unaffected land was less fragmented and represented a physical barrier to the expansion of ‘fragile’ and ‘critical’ areas in most cases (i.e. acting as a ‘buffer’ zone). ‘Fragile’ and ‘critical’ lands were, in turn, organized in small and poorly connected patches. Over time, ‘critical’ land expanded radio-centrically, incorporating both ‘fragile’ and ‘unaffected’ areas and forming a structured network across space. ‘Unaffected’ land has been strongly fragmented, acting less effectively as a buffer to the expansion of ‘critical’ land. Displaying a spatially additive expansion, ‘fragile’ lands have in turn undergone evident processes of fragmentation. Spatial polarization in affected and unaffected areas was progressively more intense in Italy, resulting in a fractal landscape^50,60,61^.

By contrast, a more homogeneous landscape was characteristic of regions exposed to a higher level of land vulnerability. The increasing size of ‘fragile’ and 'critical' land patches and a progressive fragmentation of ‘unaffected’ patches eroded the buffering capacity of less vulnerable land^12^. The recent spread of patches exposed to intrinsic degradation processes (i.e. ‘critical’ land, considered as ‘hotspots’ of land degradation) may bring to important consequences in two directions. The increasing number of hotspots may leverage the intrinsic probability of local-scale land degradation processes^46^. At the same time, since the buffering effect of ‘unaffected’ land is supposed to be more effective on smaller (than larger) degradation hotspots, this phenomenon may bring to a self-alimenting expansion of more vulnerable land^62^.

The present study documents how the spatial balance between severely affected and unaffected land is an important trait of any Mediterranean landscape, whose dynamic equilibrium was strongly influenced by background territorial (i.e. socioeconomic and environmental) conditions^63^. In this direction, landscape metrics appeared as innovative and particularly refined indicators of vulnerability to land degradation^64^. These indicators provide an information dashboard that allows a more comprehensive assessment of landscape dynamics and the overall trajectories of change over time^65^, as summarized in Table 5. The content of this table contextualizes the empirical results of our study to broader socioeconomic dimensions, in common with other Mediterranean areas. Based on a literature review, the main drivers of landscape transformation in Italy (1960–2010), often fueling land degradation, were identified and briefly commented.

**Table 5:** An overview of the latent linkages between land degradation, changes in landscape structures, and the involved natural/anthropogenic factors in Italy by observation time and selected geographical gradient.

Gradient/Factor	Early-1960s	Early-1990s	Early-2010s
North–South gradient	Environmental disparities between northern/central and southern regions; crucial role of climate aridity^66^	A marked environmental gap along the north–south gradient, with significant influence of economic development^38^	North–south environmental divides decline, with economic growth and urbanization involving Southern Italy^53^
Elevation	Vulnerable areas concentrate in economically advanced flat districts^23^	Vulnerable areas concentrated in lowlands and uplands^6^	Marked environmental disparities along elevation, strengthening the role of crop intensification and urbanization^54^
Coastal-inland	Coastal (tourism) districts include the most vulnerable land to degradation^24^	Following urbanization and economic development, land vulnerability increases inland^40^	Urbanization and infrastructure development reduce disparities between coastal and inland districts^46^
Urban–rural	Moderate environmental disparities observed along the urban–rural gradient^12^	Increasing vulnerability of peri-urban land to degradation^6^	Marked environmental disparities along the urban gradient driven by dispersed urbanization^11^
'Rurality degree'	Land vulnerability differs mostly between intensive (more vulnerable) and marginal (less vulnerable) rural systems^49^	The level of land vulnerability increases with crop intensification^55^	The level of land vulnerability increases with depopulation and abandonment of cropland in marginal districts^50^
Intrinsic vulnerability factors	Territorial disparities in land quality depend on high (or low) quality soils^47^	Natural factors (climate and soils) play a major role in generating territorial disparities in land vulnerability^9^	A complex interaction among soil, vegetation cover, and human pressures shapes disparities in affected and non-affected land^43^
Vulnerability level	Spatially-balanced distribution of 'critical', 'fragile', and 'non-affected' land; moderate impact of urbanization and agriculture on degraded areas, greater importance of climate regime^10^	Sharp increase in the extent of 'critical' land driven by urban growth, economic development and crop intensification^51^	Expansion of 'critical' areas strengthens spatial polarization in vulnerable and non-vulnerable land; stable role of urbanization, industrialization, tourism development and crop intensification^52^

The empirical results of our study allow for an operational use of landscape indicators (and literature information) from an integrated policy perspective. In Italy, the National Action Plan against Desertification (NAP) coordinates the implementation of Regional Action Plans (RAPs), which can largely benefit from the quantitative information presented in our work. In particular, landscape metrics offer a multivariate reading of vulnerable landscapes, going beyond the uni-dimensional ESAI ranking (e.g.^67,68^. The analysis of landscape metrics run in this study indicates how landscape structure was highly diversified at the regional level in Italy, likely as a response to largely differentiated (and rapidly changing) socioeconomic contexts^23,40,49,50^. In all study periods, the empirical results of the analysis go beyond the traditional dichotomy between Northern (unaffected) and Southern (affected) regions, highlighting a more heterogeneous territorial framework that mixes Southern and Northern regions as a function of changes in the dominant landscape^52^. These results outline highly differentiated levels of land vulnerability in both Northern and Southern Italy, indicating that the general strategy of the NAP (concentrating efforts to mitigate and adapt to the risk of desertification in affected areas of Southern Italy) needs a thorough revision^48^. More specifically, it is necessary to re-evaluate the classification in affected and unaffected areas, acquiring more information at a disaggregated territorial level, considering together structure, composition, configuration, and functions of vulnerable and non-vulnerable landscapes^47,69,70^. Based on landscape metrics and their empirical relationship with the ESAI, some regions of Southern Italy (Sardinia, Sicily, Apulia) and Northern Italy (Emilia Romagna, Veneto) shared high levels of vulnerability to land degradation and therefore, they can benefit from specific strategies aimed at mitigation and adaptation to global change^66^.

The multivariate analysis of landscape metrics finally demonstrated the importance of ‘unaffected’ areas as possible buffer zones containing the expansion of ‘critical’ areas^54^. These results are relevant for a 'zero net land degradation' strategy, and position ‘unaffected’ areas at the middle of integrated actions to contain the level of land vulnerability to degradation^71^. Preserving the spatial integrity and connectivity of ‘unaffected’ land therefore represents an important planning tool to mitigate desertification risk^10^. At the same time, acting preventively on the landscape mechanisms that stimulate the radio-centric expansion of ‘critical’ areas—and reducing the connectivity of ‘fragile’ areas—appear to be reasonable measures reinforcing the adaptation of local landscapes to worse environmental conditions.

## Conclusions

Our study provides a quantitative analysis of natural and anthropogenic changes affecting the level of land vulnerability to degradation in an affluent economy classified as ‘affected’ country by the United Nations Convention to Combat Desertification (UNCCD, Annex IV). By delineating non-linear trends in land vulnerability, results suggest how the spatial balance between affected and unaffected land is an important trait of any Mediterranean landscape, whose dynamic equilibrium is influenced by the background territorial conditions. A large-scale assessment based on landscape metrics may illustrate—likely better than more traditional approaches—the complex shift in landscape structure and configuration. Landscapes with more homogeneous structures and configurations are frequently associated with higher levels of land vulnerability. In other words, landscape fragmentation and diversification should be considered a positive (or negative) factor of land vulnerability depending on the specific territorial context.

Based on these premises, our work has definitely shown how the availability of large datasets with diachronic information allows a more comprehensive vision of the intimate transformations of the landscape at the basis of land degradation. This knowledge supports formulation of more targeted, place-specific planning actions counteracting the risk of desertification. Technological challenge and the growing interest in open data worldwide provides an information base of interest in this direction. At the same time, it appears increasingly necessary to make available diachronic information (e.g. from reliable data sources such as historical land-use maps) that allow a long-term assessment of landscape dynamics.
